# Real-world outcomes of spinal muscular atrophy treatment with onasemnogene abeparvovec in Croatia: a comprehensive case series and literature review

**DOI:** 10.3389/fmed.2025.1609072

**Published:** 2025-06-24

**Authors:** Ivan Lehman, Matija Matošević, Lovro Lamot, Branka Bunoza

**Affiliations:** ^1^School of Medicine, University of Zagreb, Zagreb, Croatia; ^2^Divison of Pediatric Neurology, Department of Pediatrics, University Hospital Center Zagreb, Zagreb, Croatia; ^3^Institute of Emergency Medicine of Zagreb County, Velika Gorica, Croatia; ^4^Division of Pediatric Nephrology, Dialysis and Transplantation, Department of Pediatrics, University Hospital Center Zagreb, Zagreb, Croatia

**Keywords:** onasemnogene abeparvovec, spinal muscle atrophy, CHOP INTEND, SMA type 1, gene therapy

## Abstract

**Introduction:**

The development of novel treatment options and the implementation of newborn screening programs have significantly transformed the landscape of care for patients with spinal muscular atrophy (SMA). In a relatively short span, SMA has evolved from a debilitating and fatal disorder into a treatable condition, primarily due to advancements in gene-targeted therapies. Onasemnogene abeparvovec-xioi, an adeno-associated viral vector-based gene therapy delivering a functional copy of the SMN1 gene, has shown significant efficacy in improving motor function and survival rates. In Croatia, this therapy has been integrated into routine clinical practice for several years, providing valuable real-world data on its long-term outcomes and effectiveness. The presented case study aims to document these clinical experiences, contributing to the growing body of evidence supporting the efficacy and safety of onasemnogene abeparvovec-xioi and highlighting the crucial role of early diagnosis and intervention in SMA management.

**Methods:**

We conducted a retrospective case series analysis of five pediatric patients diagnosed with SMA type 1, treated with onasemnogene abeparvovec-xioi at a tertiary care center in Croatia. Four patients presented with hypotonia and motor developmental delay, and one was identified through newborn screening. All patients had genetically confirmed SMA, underwent CHOP-INTEND (Children’s Hospital of Philadelphia Infant Test of Neuromuscular Disorders) testing pre- and post-treatment, and were monitored for clinical response and adverse events. In addition, a systematic literature search was conducted using PubMed and Scopus databases to identify reports of pediatric SMA type 1 patients treated with onasemnogene abeparvovec. Keywords used included “onasemnogene abeparvovec” and “spinal muscular atrophy.” A total of 33 articles, describing 408 pediatric patients, were included.

**Case report:**

We describe a series of five patients, four of which initially presented with varying degrees of hypotonia and delay in motor development, while one patient was discovered through newborn screening program. All patients received genetic confirmation of SMA, underwent Children’s Hospital of Philadelphia Infant Test of Neuromuscular Disorders (CHOP-INTEND) testing and received onasemnogene abeparvovec treatment. Four out of five patients achieved adequate clinical improvement as show by the increase in CHOP-INTEND score. One patient showed signs of regression and required additional care.

**Conclusion:**

Despite the widespread use of novel treatment modalities that have drastically improved patient outcomes, there remains a paucity of real-world case reports documenting the care of SMA patients. This case series and accompanying literature review reinforce the efficacy and safety of onasemnogene abeparvovec in the treatment of SMA type 1, particularly when initiated early. In addition, our case series emphasize the critical role of newborn screening in identifying affected individuals before the onset of irreversible motor neuron loss as well as prompt start of treatment in all patients. We hope that our findings will contribute meaningfully to the expanding body of literature and knowledge on spinal muscular atrophy, ultimately fostering better patient care and outcomes.

## Introduction

Spinal muscular atrophy (SMA) is an autosomal recessive neurodegenerative disorder caused by mutations in the survival motor neuron 1 (*SMN1*) gene, which is located in the 5q13 region of chromosome 5, leading predominantly to the loss of lower motor neurons and cranial nerve nuclei in the brainstem, and resulting in progressive muscle weakness and atrophy (1–3). Due to the diverse clinical manifestations and varying age groups affected, SMA is clinically classified into five distinct types, based on the age of onset, the highest motor milestones achieved and life expectancy (Supplementary Table 1) (1–4). The most common type, SMA type I, typically presents during the first 6 months of age with hypotonia, delayed motor milestones, feeding impairment, inability to sit independently, and respiratory muscle involvement. This type affects almost half of SMA patients, usually resulting in death before age of two (2–4). The pathophysiological mechanism of SMA involves biallelic loss-of-function mutations in the SMN1 gene, predominantly due to a homozygous deletion of exon 7, which leads to the production of insufficient and nonfunctional SMN protein. The *SMN2* gene, which shares high homology with SMN1, plays a contributory role in the pathogenesis of SMA since it can occasionally retain exon 7 during transcription, resulting in the production of full-length SMN protein that partially compensates for the deficits caused by *SMN1* mutations (1–3, 5). Because of that unique property, *SMN2* copy number plays a significant role in determining phenotype severity (2, 3, 5).

Until recently, the management of SMA primarily centered on supportive care, which encompassed respiratory and nutritional support in conjunction with physical therapy. In 2016 and 2017, the approval of nusinersen, a modified antisense oligonucleotide, opened new avenues for the management of SMA (6). Subsequently, two additional pharmacotherapies, onasemnogene abeparvovec-xioi and risdiplam, were introduced in 2019 and 2020 respectively, further expanding the therapeutic landscape (7, 8). Onasemnogene abeparvovec (OA), an adeno-associated viral vector-based gene therapy, developed to carry a functional copy of the gene encoding full-length SMN protein directly to motor neuron, was the first gene therapy approved as treatment for SMA in the United States (7). Soon after, Croatian Institute for Health Insurance, following European Medicine Agency approved the use of OA in Croatia, making a step forward in providing advanced treatment options for SMA patients in the country, vast majority of which are treated in a single referral center for pediatric neuromuscular disorders.

The objective of our manuscript is to evaluate the long-term efficacy and tolerability of onasemnogene abeparvovec (OA) in five patients with a genetic diagnosis of spinal muscular atrophy. This evaluation is complemented by a comprehensive systematic literature review, aiming to enhance the robustness and contextualization of our findings within the broader scientific framework.

## Methods

### Patients

Five pediatric patients (two males and three females) genetically confirmed to have biallelic SMN1 gene mutations with either 2 or 3 SMN2 gene copies and followed at the Division of Pediatric neurology, Department of Pediatrics, University Hospital Center Zagreb were included. All the patients received OA at a dose of 1.1 × 10^14^ vector genomes per kilogram of body weight between October 2021 and February 2023. Four patients were diagnosed clinically with confirmation through genetic testing. One pre-symptomatic patient was identified via the national newborn screening program in Croatia, which commenced on March 1, 2023.

### Ethical approval and consent

Informed written consent was obtained from the parents of all patients following comprehensive counseling regarding the potential risks and benefits of OA treatment and alternative therapeutic options.

### Treatment protocol

In alignment with the treatment protocol, all patients were administered prednisone at the equivalent of oral prednisolone dose of 1 mg per kilogram of body weight daily, starting 24 h prior to OA administration and continuing for approximately 30 days. Prednisolone was subsequently tapered over an additional 30-day period.

### Clinical and laboratory assessments

Standardized clinical assessments appropriate for the patients’ ages were conducted by physiotherapists using the Children’s Hospital of Philadelphia Infant Test of Neuromuscular Disorders (CHOP-INTEND). Comprehensive laboratory evaluations were performed before and after OA therapy. These evaluations included complete blood cell counts, liver function tests (total bilirubin, aspartate aminotransferase [AST], alanine aminotransferase [ALT]), coagulation profiles, and cardiac tests (troponin-I and/or troponin-T levels). Detailed patient data are presented in Table 1.

**Table 1:** Patient characteristics including initial number of SMN2 copies and CHOP-INTEND score compared with CHOP-INTEND score following treatment.

Pt. no.	SMN2 copies	Initial CHOP-INTEND score	Pretreatment	CHOP-INTEND post treatment	Follow-up time
1	two	28	No	36	18 mo
2	two	36	No	55	27 mo
3	three	25	Yes, nusinersen	55	24 mo
4	two	25	No	48	12 mo
5	three	64	No	62	10 mo

## Systemic literature search

**Figure 1:**
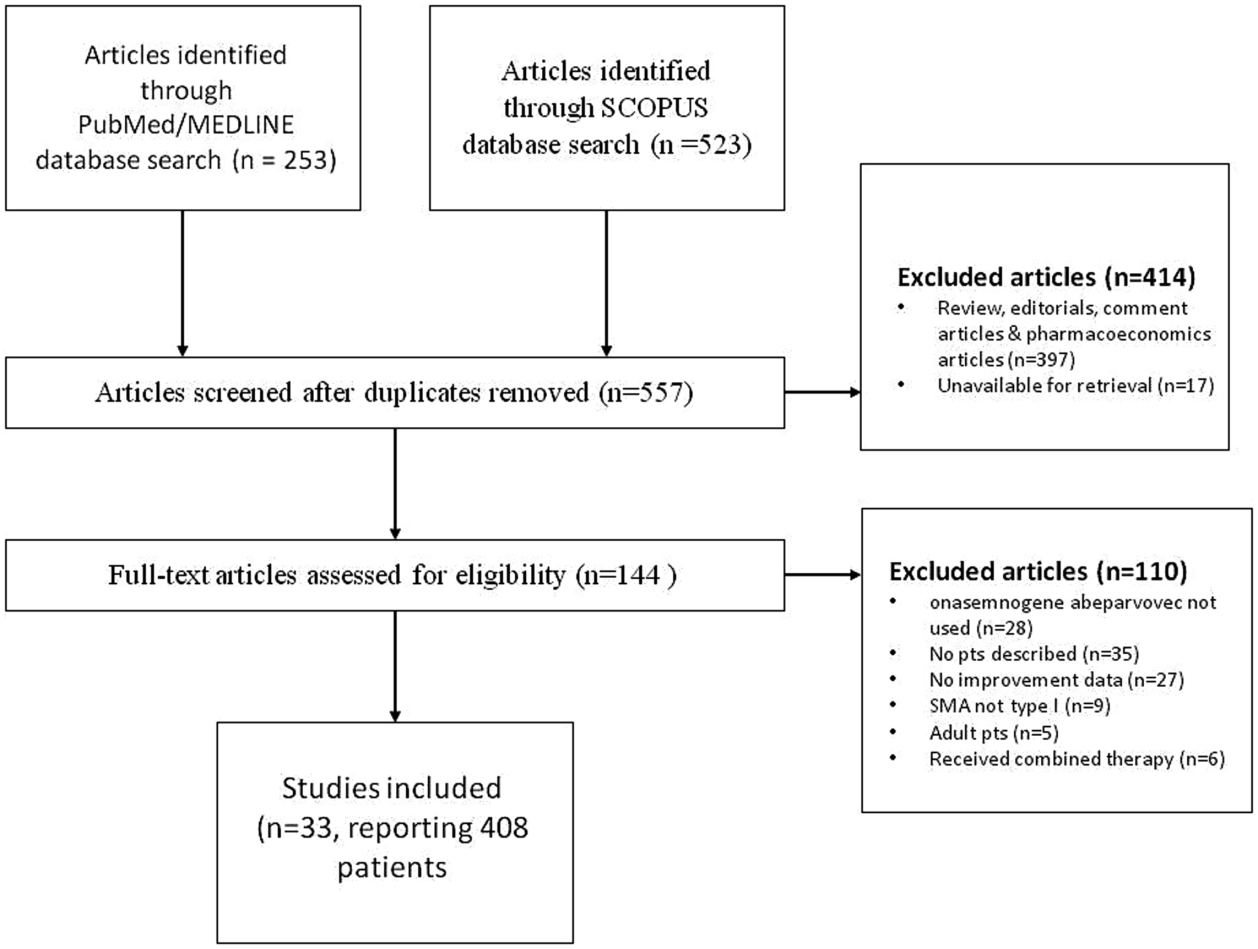
PRISM flowchart of literature review.

A systematic literature search of PudMed and Scopus database was performed to identify pediatric patients with spinal muscular atrophy type 1 that were treated with onasemnogene abeparvovec. The search was conducted by entering the keywords “onasemnogene abeparvovec” and “spinal muscular atrophy” with no time or language constraint, in accordance with the published guidance on narrative reviews. Initial search of both SCOPUS and PubMed/MEDLINE databases yielded 776 articles. In the first round of exclusions, after reviewing all the abstracts, reviews, editorials, comment and pharmacoeconomics articles were excluded as well as articles that were unavailable for retrieval. We assessed 144 full-text articles and further excluded articles that did not describe the use of onasemnogene abeparvovec, articles that contained no patient descriptions, where patients did not have SMA type 1 and were adults. We also excluded articles that contained no data pertaining to the improvement of the patients described. Our search produced 33 papers, describing 408 pediatric patients diagnosed with spinal muscular atrophy type 1 treated with onasemnogene abeparvovec. Details of selected papers can be found in Table 2, while PRISM CHART is depicted in Figure 1.

**Table 2:** Condensed overview of notable findings, outcomes and adverse effects in patients treated with onasemnogene abeparvovec gene replacement therapy for SMA type 1 discovered through literature review.

Authors	No. of PTS	Evaluation tool	Clinical outcome	Follow-up duration	Adverse effects
Al-Zaidy et al. (27)	12	CHOP-INTEND, BSID-III, WHO-MGRS	Improvement	24 months	Yes
Costamagna et al. (28)	1	CHOP-INTEND	Improvement	6 months	Yes
Nigro et al. (10)	1	HINE	Improvement	20 months	Unknown
Mizuno et al. (29)	2	CHOP-INTEND	Improvement	24 months	Unknown
Yazaki et al. (30)	1	CHOP-INTEND, HINE	Improvement	12 months	Yes
Matesanz et al. (31)	3	CHOP-INTEND, HMFSE	Improvement	12 months	Unknown
Gagliardi et al. (11)	3	CHOP-INTEND	Improvement	Up to 17 months	Unknown
Gowda et al. (32)	99	CHOP-INTEND, HINE, RHS	Improvement	Unknown	Yes
Artemyeva et al. (33)	31	CHOP-INTEND, HINE	Improvement	6 & 12 months	Yes
Sawada et al. (34)	1	CHOP-INTEND	Improvement	Unknown	Unknown
Waldrop et al. (12)	12	CHOP-INTEND, BSID, HINE, AIMS, RHS	Improvement	Up to 4 months	Yes
Ali et al. (14)	7	CHOP-INTEND	Improvement	Up to 10 months	Unknown
Al-Zaidy et al. (35)	12	BSID	Improvement	24 months	Unknown
Alves et al. (36)	1	BSID-III, WHO-MM 10s, WHO-MM	Improvement	Every 3 months	Unknown
Kokorina & Nikitin (37)	3	CHOP-INTEND	Improvement	3 for Pt1 and 10 months for Pt2 and 3	Yes
Kato et al. (38)	2	CHOP-INTEND	Improvement	Unknown	Unknown
Hale et al. (39)	6	Test not specified	Improvement	Unknown	Unknown
Bitetti et al. (40)	12	CHOP-INTEND	Improvement	12 months	Unknown
Nambu et al. (41)	1	CHOP-INTEND	Improvement	Unknown	Yes
Pitarch Castellano et al. (42)	1	CHOP-INTEND	Improvement	2 months	Unknown
Strauss et al. (9)	14	CHOP-INTEND, BSID, WHO-MGRS	Improvement	Up to 18 months	Yes
Day et al. (43)	22	CHOP-INTEND, BSID	Improvement	Until 18 months of age	Yes
D’Silva et al. (44)	16	CHOP-INTEND, WHO-MM, HFMSE	Improvement	2–26 months, median of 16	Yes
Pane et al. (13)	44	CHOP-INTEND	Improvement	6 & 12 months	Yes
Nanri et al. (18)	1	CHOP-INTEND	Improvement	24 months	Yes
Favia et al. (45)	8	CHOP-INTEND	Improvement	Up to 38 months	Unknown
Toro et al. (46)	11	Test not specified	Improvement	Up to 26.5 months	Unknown
Servais et al. (17)	70	CHOP-INTEND	Improvement	Up to 40 months	Yes
Bitetti et al. (47)	1	CHOP-INTEND, PEDI test	Improvement	Up to 36 months	Yes
Hammond et al. (48)	1	Test not specified	Improvement	16 months	Unknown
Tosi et al. (49)	1	CHOP-INTEND, HFMSE	Improvement	9 months	Yes
Lee et al. (50)	2	CHOP-INTEND	Improvement	3 months	Unknown
Stettner et al. (51)	6	CHOP-INTEND	Improvement	13 ± 3,7 months	Yes

CHOP-INTEND, Children’s Hospital of Philadelphia Infant Test of Neuromuscular Disorders; BSID-III, Bayley Scales of Infant and Toddler Development III edition; WHO-MGRS, World Health Organization Multicenter Growth Reference Study, WHO-MM, World Health Organization Motor Milestones; HINE, The Hammersmith Infant Neurological Examination; RHS, Revised Hammersmith Scale; HFMSE, Hammersmith Functional Motor Scale Expanded; AIMS, Abnormal Involuntary Movement Scale.

## Case report

### Patient 1

Patient 1 (F) manifested the first signs of hypotonia at the age of 3 weeks and was diagnosed with SMA type 1 with two copies of SMN2 at the age of 3 months. At that age she presented with severe truncal and limb hypotonia and intercostal muscle weakness. Non-invasive ventilation (NIV) was used for 5 h during sleeping. Her initial CHOP-INTEND score was 28/64. She was treated with OA at the age of 116 days. Two days after the treatment, she developed vomiting and diarrhea. Liver enzymes (AST and ALT) were elevated, but not over 2x ULN, at the end of the first week, with preserved liver function. The lowest platelet count was 47 at the end of the first week. The corticosteroid dose was increased to 2 mg/kg of prednisone for 10 days. CHOP-INTEND scores measured after OA treatment were 31, 34, 36 and 38 at 3, 6, 12 and 16 months follow-up, respectively. 11 months after receiving OA, she developed a severe respiratory infection, which required tracheotomy and mechanical ventilation. Following the resolution of infection, only nighttime mechanical ventilation was required. At 18 months, the follow-up CHOP-INTEND score showed a decline, with 36 points. She developed motor deterioration with swallowing difficulties and food aspiration as well as chest deformation. She did not meet motor development milestone of sitting without support. Eventually, add-on therapy with risdiplam was introduced at the age of 28 months. At the age of 42 months she is being fed via percutaneous endoscopic gastrostomy (PEG), is mechanically ventilated during the night and has scoliosis and poor head control.

### Patient 2

Patient 2 (F) was diagnosed with SMA type 1 with two copies of SMN2 at the age of 3 months. She initially presented at the age of 2 months with hypotonia, inability to raise upper and lower extremities upward, no head control and inability to raise head in prone position. Her initial CHOP-INTEND score at the age of 3 months was 36/64. She was treated with OA at age of 142 days. The patient was febrile the second day and vomited for 5 days. Liver enzymes were elevated, highest on day 4, AST 3.8xULN and ALT 2.8x ULN. She developed thrombocytopenia with the lowest platelet count 50 on day 7. The corticosteroid dose was increased to 2 mg/kg of prednisone. At her 1-month follow-up, her CHOP-INTEND score was 43. This upward trend continued at subsequent follow-ups at 3, 6, 12 and 24 months, with score being 44, 49, 54 and 55 points, respectively. She started to sit at the age of 16 months. At the last visit at 43 months of age, she attained sitting for 5 s without support, head control in sitting position but no control in traction. She was also able to keep legs flexed at knees, perform abduction and adduction and elevate the hands to the level of the ears. Bulbar movements were normal, but facial muscles showed mild weakness without fasciculations. She developed severe thoracic scoliosis. Parents later started risdiplam treatment at 23 months at their own expense.

### Patient 3

Patient 3 (M) was diagnosed with SMA type 1 with three copies of SMN2 at the age of 7 months after initially presenting with signs of hypotonia at 2 months of age. He was primarily treated with 4 doses of nusinersen, with the last dose being at the age of 11 months, after which OA treatment was initiated at 430 days of age. In the moment of OA administration, he was able to sit with support but without head control in traction. He was able to raise his upper extremities but could not raise his lower extremities. He was febrile and vomited in the first week after the drug application. He showed elevation of liver enzymes, with peak being at 5 days after treatment. AST was 4.7xULN and ALT 3.6xULN with preserved liver function. Platelets were lowest on the same day, measuring at 99. After that, laboratory results returned to normal values after 2 weeks. Eventually, corticosteroids were tapered off after 3 months. Four months after application there was a second elevation of liver enzymes (AST 1.6xULN and ALT 2.9xULN), and corticosteroids were introduced again for the next 3 months. CHOP-INTEND score pre-OA treatment was 25 at 8 months of age, before nusinersen treatment and 45 at 14 months after nusinersen treatment. The positive trend of CHOP-INTEND score increase continued, with the scores being 47, 50, 53 and 55 at 3, 6, 12 and 24-month follow-ups. He started to sit independently at the age of 20 months. Neurological exam at 2 years old showcased ability to sit without support for more than a minute and raise upper extremities above shoulder level. He also exhibited thoracic kyphosis and thoracolumbar scoliosis. He was able to stand with support and push wheelchair by himself.

### Patient 4

Patient 4 (M) was diagnosed with SMA type 1 with two copies of SMN2 gene at the age of 2 months after presenting with progressive hypotonia and weakness at 1 month of age, tongue fasciculation and weakness of intercostal muscles with areflexia. Initial CHOP-INTEND score was 20. He received OA at the age of 67 days. Granulocytopenia with positive antigranulocyte antibodies was noticed before application OA with ANC of 0.58 that got more severe during the first week after drug application, with the lowest ANC being 0.19. Granulocytopenia resolved after 4 months. During the first week after treatment there was elevation of AST and ALT below 2xULN as well as mild thrombocytopenia of 167 that both resolved afterwards CHOP-INTEND scores were 23, 35, 43 and 48 on follow-up at 1, 3, 6 and 12 months, respectively. He started to sit independently at the age of 15 months and was able to stand with support at the age of 20 months. Language skills were developing adequately, with vocabulary consisting of few words and adequate nonverbal communication.

### Patient 5

Patient 5 (F) was diagnosed with SMA type 1 with three copies of SMN2 through newborn screening. Her initial CHOP-INTEND score was 64. She was treated as a pre-symptomatic patient and received OA at age of 26 days. During the first week there was elevation of AST and ALT below 2xULN and mild thrombocytopenia of 114 platelets. She also had an elevation of troponin I level to 128.4 ng/L on day 7 (ref. 0–15.6 ng/L) with a normal heart ultrasound and ECG. Her psychomotor development was normal and without developmental delays. She walks independently at the age of 11 months. Her social development was also normal. She did not show any signs of disease.

**Figure 2:**
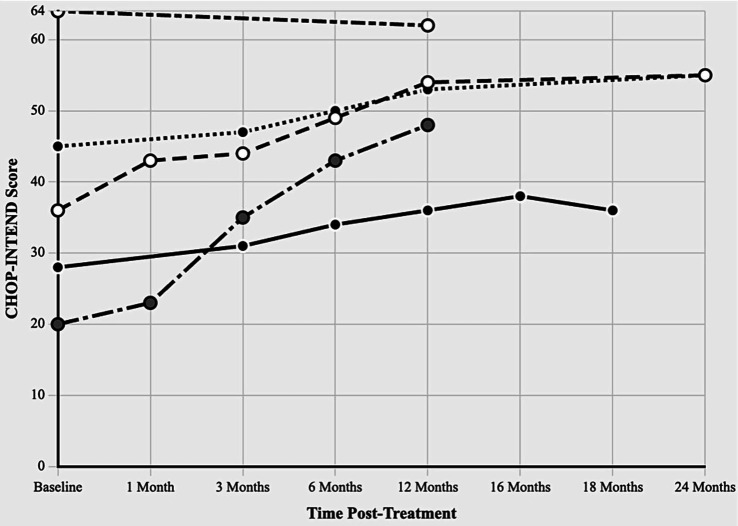
CHOP-INTEND score trajectories following onasemnogene abeparvovec treatment. Children’s hospital of Philadelphia infant test of neuromuscular disorders (CHOP-INTEND) scores from baseline through 24 months post-treatment in five Croatian SMA patients. Maximum possible score: 64 points. Different line patterns represent individual patients with varying SMN2 copy numbers and treatment ages.

The detailed characteristics and outcome of patients is showed in Table 1 while CHOP-INTEND Score Trajectories following onasemnogene abeparvovec treatment are graphically displayed in Figure 2 and Supplementary Table 2.

**Table:** 

____	**Patient 1 (Female)**: 2 SMN2 copies, treated at 116 days of age. Required tracheostomy at 11 months post-treatment. Poor clinical outcome.
- - -	**Patient 2 (Female)**: 2 SMN2 copies, treated at 142 days of age. Achieved sitting without support at 16 months. Good clinical improvement.
····	**Patient 3 (Male)**: 3 SMN2 copies, treated at 430 days of age. Prior nusinersen treatment. Achieved sitting and standing with support.
-·-·	**Patient 4 (Male)**: 2 SMN2 copies, treated at 67 days of age. Achieved sitting (15m) and standing (20m) with support. Best improvement (+140%).
-··-	**Patient 5 (Female)**: 3 SMN2 copies, treated at 26 days of age. Presymptomatic treatment via newborn screening. Normal development, independent walking at 11 months.

## Results

Our literature review produced a selection of case reports and studies pertaining to treatment of children with SMA type 1 using onasemnogene abeparvovec for comparison (Supplementary Table 3). The number of patients in every paper noted in our literature review varied greatly, from single case reports to multicenter studies done in university clinical centers. Most commonly used evaluation tool was CHOP-INTEND, often paired with other tests such as The Bayley Scales of Infant and Toddler Development III edition (BSID-III), World Health Organization Multicenter Growth Reference Study (WHO-MGRS), World Health Organization Motor Milestones (WHO-MM) The Hammersmith Infant Neurological Examination (HINE), Revised Hammersmith Scale (RHS), Hammersmith Functional Motor Scale Expanded (HFMSE) and Abnormal Involuntary Movement Scale (AIMS) In two papers, by Hale et al. and Hammond et al., evaluation tools for observing motor milestones and motor development of the patients were not specified. All patients showed clinical improvement upon receiving treatment with OA. The follow-up duration varied greatly, ranging from 2 months up to 40 months. Seventeen out of 33 papers reported some kind of adverse effect with most prevalent being pyrexia and transaminases increase.

## Discussion

We presented a single center retrospective cohort analysis of real-world data in treatment of children with SMA type 1 using onasemnogene abeparvovec. In our cohort all children showed clinical improvement, as shown by the increase in CHOP-INTEND score during follow-up visits. Nevertheless, one patient eventually showed deterioration of motor and bulbar functions and a need for mechanical ventilation during sleep. The best outcome of treatment was exhibited by our patient who was diagnosed through a newborn screening program and has three copies of SMN2 gene, which enabled us to treat her at the age of 26 days, before the manifestation of clinical signs of disease. The child reached the milestone of walking independently at the appropriate age. This is comparable with studies showcasing treatment in pre-symptomatic patients within the first weeks of life, which results in favorable or even normal motor function in children with SMA (9–14). Considering that early interventions result in better outcome, a significant step forward in the direction of early treatment was achieved with implementation of newborn screening (15, 16). Even though newborn screening is relatively new in Croatia, our patient greatly benefited from early treatment that was undertaken due to the screening program. Our one patient identified through newborn screening was born in 2023, coinciding with the implementation of SMA newborn screening in Croatia. The remaining four patients were born prior to 2023 and were diagnosed based on clinical presentation. Since the establishment of newborn screening in Croatia in 2023, the program has identified 10 new SMA cases nationally. We deliberately excluded additional newborn screening-identified patients from this case series due to insufficient follow-up duration to assess meaningful clinical outcomes. However, we agree that expanding newborn screening programs internationally represents a critical public health priority, as evidenced by the superior baseline status of our screening-identified patient.

Four out of five children, three of which were diagnosed with SMA type 1, reached the milestone of sitting independently. Similar results of almost 50% or more patients achieving this milestone were presented in studies by Strauss et al. and Servais et al. respectively (9, 17). Patient 3, with three SMN2 copies received four doses of nusinersen prior to beginning treatment with OA at the age of 14 months and continued to improve after the switch. Only he had a second elevation of aminotransferase after discontinuation of corticosteroid treatment. Patient 1 did not achieve a CHOP-INTEND score greater than 40. In addition, she also required tracheostomy 11 months after treatment and mechanical ventilation during the night. It is a child with SMA type 1a who had severe symptoms at the age of 2 months and was severely affected by infection at the age of 11 month. Risdiplam therapy was initiated at the age of 28 months after the deterioration of bulbar motor functions. Combined therapy of nusinersen with OA and risdiplam with OA might have some benefits and is considered safe (18–21). Regarding patient 2, the parent opted for combined therapy of onasemnogene abeparvovec and risdiplam which they procured on their own expense. Due to the popularity of crowdfunding platforms such as GoFundMe or Kickstarter, a viable option for acquiring monetary means for treatment is a crowdfunding campaing (22). However, these types of funding platforms are still relatively unpopular in Croatia, with parents usually depending on their own income or funds raise through various charity organizations and events.

OA is mostly well tolerated in both pre-symptomatic and symptomatic patients, with adverse effect usually consisting of pyrexia, vomiting, loss of appetite and transaminases increase (9, 13, 14, 17). All our patients had an elevation of transaminase levels without liver function deterioration and drop in platelet count without clinical significance. These adverse effects were transitory. One patient had worsening of autoimmune granulocytopenia. In patient 3, who was the oldest at the time of treatment and who was previously treated with nusinersen, there was another elevation of transaminases after discontinuation of corticosteroid therapy that resolved with restarting of corticosteroid therapy. Patient 5 had transitory asymptomatic troponin I elevation. It is important to mention that OA administration can cause some serious adverse events, including hepatic failure and thrombotic microangiopathy, as well as an increase in heart enzymes levels such as troponin-I and troponin-T (23–26).

## Conclusion

Spinal muscular atrophy (SMA) poses a significant healthcare challenge due to its progressive nature and the diverse pediatric population it affects. Prior to the introduction of disease-modifying treatments, there was no effective way to combat this debilitating disease. This case series highlights the complexities and urgency involved in decision-making for treating a life-threatening condition like SMA, where timely intervention can critically impact outcomes. The intricate nature of the disease and its treatments necessitates careful consideration of the most appropriate therapy for each patient. Consequently, case reports such as ours are invaluable, providing insights and education for healthcare providers on various treatment and follow-up approaches. These contributions enrich the existing literature and support informed decision-making in the management of SMA.

## Data Availability

The original contributions presented in the study are included in the article/Supplementary material, further inquiries can be directed to the corresponding author.
