# Supplementary material for: Real-world outcomes of spinal muscular atrophy treatment with onasemnogene abeparvovec in Croatia: a comprehensive case series and literature review
Source: Front Med (Lausanne). 2025 Jun 24;12:1609072. doi: 10.3389/fmed.2025.1609072 (PMC12234475; doi:10.3389/fmed.2025.1609072)
Supplement: Supplementary file 1 [file Table_1.DOCX]

**Supplementary Table 1. Classification of spinal muscular atrophy, modified according to Arnold and Fischbeck, Nicolau et al, Keinath et al and Nishio et al** (2–5)

| **Type** | SMN2 copies | Onset | Motor milestones | Clinical presentation | Life Expectancy* | Proportion of patients |
| --- | --- | --- | --- | --- | --- | --- |
| **0** | 1 | At birth | Unable to sit, no head control; respiratory failure at birth | Generalized weakness with hypotonia, contractures and respiratory and feeding difficulties | Few weeks | <1% |
| **1** | 2-3 | Between birth and 6 months | Unable to sit | Predominantly proximal weakness, tongue fasciculations, respiratory distress and poor feeding | Death by age 2 | 45-60% |
| **2** | 2-4 | 6 to 18 months | Can sit, unable to stand or walk unsupported | Predominantly proximal weakness, tongue fasciculations and scoliosis | Up to 25 years | 20-30% |
| **3** | 3-5 | Between 18 months and 30 years | Can stand and walk unsupported | Abnormal gait due to predominantly proximal weakness of lower extremities | Normal | 10-30% |
| **4** | 3-5 | >30 years | Can stand and walk unsupported | Ability to walk unsupported maintained | Normal | <5% |

*without mechanical ventilation nor disease-modifying treatment
