# Supplementary material for: Real-world outcomes of spinal muscular atrophy treatment with onasemnogene abeparvovec in Croatia: a comprehensive case series and literature review
Source: Front Med (Lausanne). 2025 Jun 24;12:1609072. doi: 10.3389/fmed.2025.1609072 (PMC12234475; doi:10.3389/fmed.2025.1609072)
Supplement: Supplementary file 2 [file Table_2.DOCX]

Supplementary Table 2. Enhanced Patient Characteristics and Outcome Summary

| **Patient** | **Sex** | **SMN2 copies** | **Treatment Age (days)** | **Pre-treatment Therapy** | **CHOP-INTEND SCORES** | | | **Motor Milestone** | | | **Peak Adverse Events** | | | | **Follow-up Duration** | **Overall Outcome** |
| --- | --- | --- | --- | --- | --- | --- | --- | --- | --- | --- | --- | --- | --- | --- | --- | --- |
|  |  |  |  |  | **Baseline** | **Best Score** | **Change** | **Sitting** | **Standing** | **Walking** | **AST**  **(xULN)** | **ALT**  **(xULN)** | **Plt (×10^3^/μL)** | **Other** |  |  |
| **1** | F | 2 | 116 | None | 28 | 38 | **+10** | **No** | **No** | **No** | **1.8** | **1.8** | **47** | **Vomiting Diarrhea** | **18m** | **Poor – Required tracheostomy** |
| **2** | F | 2 | 142 | None | 36 | 55 | **+19** | **Yes (16m)** | **No** | **No** | **3.8** | **2.8** | **50** | **Fever Vomiting** | **27m** | **Good improvement** |
| **3** | M | 3 | 430 | Nusinersen* | 45*** | 55 | **+10** | **Yes** | **Yes** | **No** | **4.7** | **3.6** | **99** | **Fever Vomiting** | **24m** | **Good improvement** |
| **4** | M | 2 | 67 | None | 20 | 48 | **+28** | **Yes** | **Yes** | **No** | **1.5** | **1.5** | **167** | **Granulocytopenia** | **12m** | **Good improvement** |
| **5** | F | 3 | 26 | None** | 64 | 62**** | **-2** | **Yes** | **Yes** | **Yes** | **1.8** | **1.8** | **114** | **troponin I ↑** | **10m** | **Good improvement** |

F = female; M = make; SMN2 = Survival Motor Neuron 2; CHOP-INTEND = Children's Hospital of Philadelphia Infant Test of Neuromuscular Disorders; AST = aspartate aminotransferase; ALT = alanine aminotransferase; ULN = upper limit of normal; AE = adverse event; m = months; Plt = platelets

Notes:

*4 doses were given

** Patient was presymptomatic

***Patient 3 baseline score is post-nusinersen treatment (pre-nusinersen score was 25)

**** Patient 5 only has 10-month follow-up avaliable

Color code: Green = achieved milestone/good outcome; Red = not achieved/poor outcome, severe adverse event; Yellow = mild adverse event; Orange = moderate adverse event
