# Supplementary material for: Real-world outcomes of spinal muscular atrophy treatment with onasemnogene abeparvovec in Croatia: a comprehensive case series and literature review
Source: Front Med (Lausanne). 2025 Jun 24;12:1609072. doi: 10.3389/fmed.2025.1609072 (PMC12234475; doi:10.3389/fmed.2025.1609072)
Supplement: Supplementary file 3 [file Table_3.DOCX]

Supplementary Table 3. Notable findings, outcomes and adverse effects in patients treated with onasemnogene abeparvovec gene replacement therapy for SMA type 1.

| **AUTHORS** | **NO. OF PTS** | **EVALUATION TOOL** | **CLINICAL OUTCOME** | **FOLLOW-UP DURATION** | **ADVERSE EFFECTS** |
| --- | --- | --- | --- | --- | --- |
| Al-Zaidy et al(1) | 12 | CHOP-INTEND, BSID-III, WHO-MGRS | All infants that received treatment maintained clinically significant improvement (CHOP-INTEND score >= 4.0 points gained); 11 of 12 pts scored >= 40.0 points in CHOP-INTEND | 24 months | 3 pts had side-effects attributed to treatment; asymptomatic elevation of serum aminotransferase |
| Costamagna et al (2) | 1 | CHOP-INTEND | 4 weeks postinfusion CHOP score was 32 points (up from 22 before treatment); at 6 months follow-up patient showcased moderate head control, improved hypotonia, increas in lower limb strenght and hand prehension; preserved spontanious breathing and swallowing; sitting without assistance for 5 seconds; CHOP score at 6 months was 42 | 6 months | Transient elevation of AST; no other side effects were noted |
| Nigro et al (3) | 1 | HINE | Head control at 4 montsh, independent walking at 18 months; pulling to stand, walk and sit independantly at 20 months, independantly reaching for objects, swallowing and breathing normal, knows up to 10 words; EMG study nwith no signs of denervation | 20 months | Not disclosed |
| Mizuno et al (4) | 2 | CHOP-INTEND | Pt1 - CHOP score 18 at 2 months (before nusinersen), 37 at 6 months (before OA), 46 at 1 year (6 months after OA), 58 at 2 years (18 months after OA); Pt2 - CHOP score 15 at 6 months (before nusinersen), 16 at 7 months (before OA), 43 at 1 year (5 months after OA) and 49 at 2 years (17 months after OA) | 2 years (18 months after OA administration) | Not disclosed |
| Yazaki et al (5) | 1 | CHOP-INTEND, HINE | CHOP and HINE-2 score before OA at 21 points (due to nusinersen usage); at 1 year after OA, CHOP and HINE-2 scores were 51 and 21 respectively | 1 year | Thrombocytopenia, microangiopathic hemolytic anemia, liver diysfunction and elevated LDH on day 5, on dialysis after day 8; *E.coli* culture negative, ADAMTS13 activity normal; C4 and CH50 slightly decreased, C3 was normal; 4 cycles of plasmapheresis after day 9; single dose of eculizumab on day 13; systemic managment and supportive care until day 30, after that dialysis free and TMA resolved; laboratory findings normalized, discharged on day 51 |
| Matesanz et al (6) | 3 | CHOP-INTEND, HMFSE | Pt1 - 7 and 16 points on HMFSE at 3 and 12 months respectively, rolling at 19 months; Pt2 - 38 and 50 points on CHOP-INTEND at 3 and 12 months, sitting >30 sec at 9.5 months; Pt3 - 40 and 58 points on CHOP-INTEND at 3 and 12 months, sitting >30 sec at 8.5 months | 1 year | Not disclosed |
| Gagliardi et al (7) | 3 | CHOP-INTEND | Pt1 - initial CHOP-INTEND was 31/64 at 15 days, at 12 months score was 64/64, walking with support at 15 months; Pt2 - initial CHOP-INTEND 38/64, at 6 months score was 64/64, sitting with support achieved at 6.5 months; Pt3 - initial CHOP-INTEND 41/64, at 3 months score was 35/64, head control achieved at 2.5 months, | Up to 17 months | Not disclosed |
| Gowda et al (8) | 99 | CHOP-INTEND, HINE, RHS | 80% of pts showed increase in CHOP-INTEND score, indicating improvements in motor function | Not disclosed | 2/3 of the patients had asymptomatic thrombocytopenia, 34% pts had elevated troponin-I unrelated to any other relevant cardiac signs or symptomes |
| Artemyeva et al (9) | 31 | CHOP-INTEND, HINE-2 | CHOP-INTEND and HINE-2 score of 17 pts at 6 months showed increas in average score by 7,1 and 3,3 respectevely; at 12 months follow up of another 10 pts CHOP-INTEND and HINE-2 score showed increas in average score by 9,4 and 4,4 respectevely | 6 & 12 months | Adverse effects included pyrexia, decreased appetite, vomiting and short-term rash |
| Sawada et al (10) | 1 | CHOP-INTEND | Initial CHOP-INTEND score was 22, but dropped to 15 before treatment; after treatment, at 8 months of age CHOP-INTEND score was 34, did not require respiratory support, oral feeding achieved at 2 years 1 month; only developmental milestone was rolling over; pt is undergoing physical and occupational therapy | Not disclosed | Not disclosed |
| Waldrop et al (11) | 12 | CHOP-INTEND, BSID, HINE, AIMS, RHS | 9 out of 12 pts showed increase in motor function test scores after treatment, 6 out of 12 pts achieved sitting, 8 out of 12 achieved oral feeding (5 partial) and 8 out of 12 patients required BiPAP (4 at night and 2 intermittent) | Up to 4 months | 5 out of 12 patients developed fever 3 days after OA administration, 11 out of 12 had elevated AST, 9 out of 12 had elevated ALT and 4 out of 12 had elevated gGT |
| Ali et al (12) | 7 | CHOP-INTEND | 6 out of 7 described patients showed improvements in CHOP-INTEND scores (mean change 11.8, range 7-18) | Up to 10 months | Not disclosed |
| Al-Zaidy et al (13) | 12 | BSID | 11 out of 12 achieved full head control and unassisted sitting (11 sat for >5 sec, 10 for >10 sec and 9 for >30 sec during 2 year follow-up); 9 pts were able to crawl and 2 were able to crawl, oull to stand and walk independently | 2 years | Not disclosed |
| Alves et al (14) | 1 | BSID-III , WHO-MM 10s, WHO-MM | The patient was asymptomatic; achieved elicited or independent rolling, sitting independently, standing supported and walking independently; no furhter data was noted | Every 3 months | Not disclosed |
| Kokorina & Nikitin (15) | 3 | CHOP-INTEND | CHOP-INTEND scores increased from 17, 20 and 23 to 36, 41 and 33 respectevaly; all pts were bed-bound with motor skill developmental delays; Pt1 was able to hold head for up to 2 minutes; Pt2 can maintain face down positin with support on the forearms, hold the head up for 20 sec, can raise the lowered head and turn it side to side, sits readily after passive set and can stand in tutors on ankle joints without support for up to 2 minutes; Pt3 learnd to turno from back to stomach, can hold head up for an hour, can raise head | 3 for Pt1 and 10 months for Pt2 and 3 | Pt1 had elevated AST & LDH levels while Pt2 and 3 had elvated AST, ALT and LDH levels |
| Kato et al (16) | 2 | CHOP-INTEND | Sitting without support at 5 months, crawling on hands and knees at 7 months, standing with assistance at 8 months, standing unassisted and walking with assistance at 10 months, unassisted walking at 11 months, jumping at 2 years 2 months; head control achieved at 6 months, but otherwise no improvement until introduction of risdiplam, further improvement noted at 11 months | Not disclosed | Not disclosed |
| Hale et al (17) | 6 | No data | Pt1- moderate head contorl, sits unasissted, rolls over, cannot lift head from prone position, no crawling or walking, able to chew and swallow; Pt2 - crawling, sitting indipendantly, pulls to stand at 15 months, walks with a walker, oral feeder; Pt3 - at 1 year old aquires age-appropriate motor milestones; Pt4 - mild motor delay due to use of braces for hip dislocation; Pt5 - acquires improved tone and head control in prone and supported sitting position; eats pureed food af 4 months; Pt6 - bilateral lower extremities weakness | Not disclosed | Not disclosed |
| Bitetti et al (18) | 12 | CHOP-INTEND | Mean CHOP-INTEND score increased by 32.4 points for nusinersen-naïve patients and 11.2 points in pts who were pretreated with nusinersen; all pts achieved head control and were able to sit if supported, 11 of 12 pts were able to roll unassisted, 5 pts could stand with support, 5 were able to stand unassisted and walk with support | 1 year | Not disclosed |
| Nambu et al (19) | 1 | CHOP-INTEND | CHOP score increase from initial 11 to 21 after 8 days; after 5.5 months CHOP score was 30 | Not disclosed | Fever and elevated AST & ALT on day 3 |
| Castellano et al (20) | 1 | CHOP-INTEND | Pt showed increase in CHOP-INTEND score of 3 points at 1 month check-up and 7 points at 2 months check-up from initial score od 21 | 2 months | Not disclosed |
| Strauss et al (21) | 14 | CHOP-INTEND, BSID, WHO-MGRS | All pts achieved the primary endpoint of independent sitting for 30 sec at any visit up to 18 months; all pts achieved motor milestones as defined by both Bayley-III (BSID) and WHO-MGRS; 11 of 14 pts stood alone, 9 walked independently at median age of 526 days, 10 pts walked alone by WHO-MGRS criteria at median age of 493 days; all 14 pts survived and were free of permanent ventilatio at 14 months of age; all 14 pts achieved CHOP-INTEND score >40, ultimately achieving a score of at least 58 by 18 months | Up to 18 months | 3 pts had hepatotoxicity, 1 pt had elevated aminotransferase enzyme concentretions, 2 pts exhibited mild to moderate elevations of creatine phosphokinase, creatine phosphokinase-MB or troponin I; all resolved |
| Day et al (22) | 22 | CHOP-INTEND, BSID | 14 of 22 pts achieved independent sitting, 19 of 22 pts were feeding orally, baseline mean CHOP-INTEND score of 32 increased by 6.9, 11.7 and 14.6 points at 1, 3 and 6 months post treatment respectevaly; 21 pts achieved CHOP-INTEND score of >40 points, 14 achieved >50 and 5 achieved >60 points | Until 18 months of age | All pts had adverse events, most common being pyrexia; adverse events related to drug administration included aminotransferase increase and hydrocephalus |
| D'Silva et al (23) | 16 | CHOP-INTEND, WHO motor milestones, HFMSE | Average icrease of 10 points in HFMSE and 7 points in CHOP-INTEND | 2-26 months, median of 16 | Most common adverse effects related to treatment were vomiting and elevated aminotransferase concentration |
| Pane et al (24) | 44 | CHOP-INTEND | All patients achieved statisticaly meaningful increase in CHOP-INTEND score (details not disclosed for individual patient) | 6 & 12 months | Most common adverse effects included pyrexia and vomiting (details for individual patients not disclosed) |
| Nanri et al (25) | 1 | CHOP-INTEND | Overall CHOP-INTEND score improved from 26 up to 58 points; pt was able to maintain sitting position, at 12 months of age the pt started training to stand with a brace; respiratory funciton improved and resp. support was no longer required; swallowing remained inadequate, with partial reliance on NG tube | 24 months | Increase in transaminase levels was noted 1 month after treatment administration with no signs of cholestasis or liver failure; reduced after the increase of prednisolone dose |
| Favia et al (26) | 8 | CHOP-INTEND | 6 pts showed increase in CHOP-INTEND scores by 2, 4, 9, 10, 17 and 31 points respectavely, 1 pt had no change in scoring while 1 pt had a 3 point drop in CHOP-INTEND score; 3 out of 8 pts maintainde head control, 5 out of 8 maintained sitting position for 30 sec unassisted | Up to 38 months | Not disclosed |
| Toro et al (27) | 11 | Test not specified | Pts that received OA had numerically greater percentag of motor milestone achievments; achievments not specified for individual pt | Up to 26.5 months | Not disclosed |
| Servais et al (28) | 70 | CHOP-INTEND | Data is avaliable for 8 pts identified through newborn screening and 19 pts identified through clinical diagnosis; CHOP-INTEND score increased from mean 44.9 to 57.0 for NBS group and 37.4 to 52.1 for clinical diagnosis group | Up to 40 months | Data is not shown for the specific pts; overall advesre effect occurred during short-term post-infusion pediod and included hepatotoxicity, transient thrombocytopenia, cardiac AE and TMA |
| Bitetti et al (29) | 1 | CHOP-INTEND, PEDI test | CHOP-INTEND increrased from 11 to 64 points; sitting with support at 24 months, sitting without support at 27 months; improvement of bulbar function and mostly oral semisolid feeding with no respiratory difficulties (after adding risdiplam) | Up to 36 months | Moderate to sever thrombocytopenia normalized within 2 weeks; transaminase levels increased in 3rd week after OA treatment but normalized after prednisolone dosage adjustment |
| Hammond et al (30) | 1 | Test not specified | At 18 months severe motor delay is present; pt attained neck control but requires support while sitting; NG tube placement is scheduled; nocturnal BiPAP is needed | 16 months | Not disclosed |
| Tosi et al (31) | 1 | CHOP-INTEND, HFMSE | CHOP-INTEND increased from 32 to 59 points; HFMSE score was 18/66; pt had stable sitting position, ability to maintain kneeling position with anterior support; could stand unaided with upper limb support; fed by mouth, no swallowing impairment, no respiratory impairment | 9 months | Fever and loss of appetite two days after infusion; five days after infusion lab results showed elevated levels of ALT and AST as well as thrombocytopenia and increased ferratin levels; all gradually resolved after doubling the dose of prednisolone |
| Lee et al (32) | 2 | CHOP-INTEND | Pt1 - initial CHOP-INTEND score was 40/64 but due to waiting for OA administration, score went down to 26; 3 months after treatment score was 43/64, problems regarding feeding and intermittent tachypnea were present; pt has not achieved rolling or independent sitting; Pt2 - initial CHOP-INTEND was 43; 3 months after OA treatment score went up to 58/64; normal feeding and growth with no respiratory issues | 3 months | Not disclosed |
| Stettner et al (33) | 6 | CHOP-INTEND | Mean baseline CHOP-INTEND score was 26.3 +/- 9.9; after OA treatment score was 49.2 +/- 7.2 (increase of mean CHOP-INTEND score of 28.1); 5/6 pts achieved head control and ability to roll onto the side; 3/6 pts were able to sit; none pts achieve the ability to crawl, stand or walk with or without support; all pts developed usefull hand function with ability to raise the hands to the mouth and 3/6 pts were able to raise hands above their heads | 406 ± 113 days | All pts exhibited transient thrombocytopenia and fluctuations in AST and/or ALT levels; 5/6 pts were hospitalized, 4 for elective and 2 for acute reasons |

CHOP-INTEND - The Children’s Hospital of Philadelphia Infant Test of Neuromuscular Disorders, HINE - The Hammersmith Infant Neurological Examination, HMFSE - Hammersmith Functional Motor Scale – Expanded, BSID - Bayley Scales Of Infant and Toddler Development, WHO-MM – World Health Organization – Motor Milestones, AIMS - Abnormal Involuntary Movement Scale, RHS - Revised Hammersmith Scale, WHO-MGRS - The World Health Organization - Multicentre Growth Reference Study,

1. Al-Zaidy SA, Kolb SJ, Lowes L, Alfano LN, Shell R, Church KR, et al. AVXS-101 (Onasemnogene Abeparvovec) for SMA1: Comparative Study with a Prospective Natural History Cohort. *Journal of Neuromuscular Diseases*. 2019;6(3): 307–317. https://doi.org/10.3233/JND-190403.

2. Costamagna G, Govoni A, Wise A, Corti S. Bridging the Gap: Gene Therapy in a Patient with Spinal Muscular Atrophy Type 1. *Neurology*. 2022;99(21): 952–956. https://doi.org/10.1212/WNL.0000000000201294.

3. Nigro E, Grunebaum E, Kamath B, Licht C, Malcolmson C, Jeewa A, et al. Case report: A case of spinal muscular atrophy in a preterm infant: risks and benefits of treatment. *Frontiers in Neurology*. 2023;14. https://doi.org/10.3389/fneur.2023.1230889.

4. Mizuno T, Kanouchi T, Tamura Y, Hirata K, Emoto R, Suzuki T, et al. Changes in electrophysiological findings of spinal muscular atrophy type I after the administration of nusinersen and onasemnogene abeparvovec: two case reports. *BMC Neurology*. 2023;23(1). https://doi.org/10.1186/s12883-023-03420-2.

5. Yazaki K, Sakuma S, Hikita N, Fujimaru R, Hamazaki T. Child Neurology: Pathologically Confirmed Thrombotic Microangiopathy Caused by Onasemnogene Abeparvovec Treatment for SMA. *Neurology*. 2022;98(19): 808–813. https://doi.org/10.1212/WNL.0000000000200676.

6. Matesanz SE, Battista V, Flickinger J, Jones JN, Kichula EA. Clinical Experience With Gene Therapy in Older Patients With Spinal Muscular Atrophy. *Pediatric Neurology*. 2021;118: 1–5. https://doi.org/10.1016/j.pediatrneurol.2021.01.012.

7. Gagliardi D, Canzio E, Orsini P, Conti P, Sinisi V, Maggiore C, et al. Early spinal muscular atrophy treatment following newborn screening: A 20-month review of the first Italian regional experience. *Annals of Clinical and Translational Neurology*. 2024;11(5): 1090–1096. https://doi.org/10.1002/acn3.52018.

8. Gowda V, Atherton M, Murugan A, Servais L, Sheehan J, Standing E, et al. Efficacy and safety of onasemnogene abeparvovec in children with spinal muscular atrophy type 1: real-world evidence from 6 infusion centres in the United Kingdom. *The Lancet Regional Health - Europe*. 2024;37. https://doi.org/10.1016/j.lanepe.2023.100817.

9. Artemyeva SB, Papina YuO, Shidlovskaya OA, Monakhova AV, Vlodavets DV. Experience of using gene replacement therapy with Zolgensma® (onasemnogene abeparvovec) in real clinical practice in Russia. *Nervno-Myshechnye Bolezni*. 2022;12(1): 29–38. https://doi.org/10.17650/2222-8721-2022-12-1-29-38.

10. Sawada T, Kido J, Yae Y, Yuge K, Nomura K, Okada K, et al. Gene therapy for spinal muscular atrophy is considerably effective when administered as early as possible after birth. *Molecular Genetics and Metabolism Reports*. 2023;35. https://doi.org/10.1016/j.ymgmr.2023.100973.

11. Waldrop MA, Karingada C, Storey MA, Powers B, Iammarino MA, Miller NF, et al. Gene therapy for spinal muscular atrophy: Safety and early outcomes. *Pediatrics*. 2020;146(3). https://doi.org/10.1542/PEDS.2020-0729.

12. Ali HG, Ibrahim K, Elsaid MF, Mohamed RB, Abeidah MIA, Al Rawwas AO, et al. Gene therapy for spinal muscular atrophy: the Qatari experience. *Gene Therapy*. 2021;28(10–11): 676–680. https://doi.org/10.1038/s41434-021-00273-7.

13. Al-Zaidy S, Pickard AS, Kotha K, Alfano LN, Lowes L, Paul G, et al. Health outcomes in spinal muscular atrophy type 1 following AVXS-101 gene replacement therapy. *Pediatric Pulmonology*. 2019;54(2): 179–185. https://doi.org/10.1002/ppul.24203.

14. Alves CRR, Petrillo M, Spellman R, Garner R, Zhang R, Kiefer M, et al. Implications of circulating neurofilamentsfor spinal muscular atrophytreatment early in life: A case series. *Molecular Therapy Methods and Clinical Development*. 2021;23: 524–538. https://doi.org/10.1016/j.omtm.2021.10.011.

15. Kokorina AA, Nikitin SS. Interim Analysis of Treatment Outcomes of Young Children with 5q Spinal Muscular Atrophy on Gene Replacement Therapy with Onasemnogene Abeparvovec. Clinical Observations. *Voprosy Sovremennoi Pediatrii - Current Pediatrics*. 2022;21(6): 535–547. https://doi.org/10.15690/vsp.v21i6S.2497.

16. Kato T, Yokomura M, Urano M, Sato Y, Ashihara Y, Ito M, et al. Two cases of spinal muscular atrophy type I that received early treatment and achieved improved prognosis by promoting multi-center, multi-professional collaboration after prenatal diagnosis. *No To Hattatsu*. 2023;55(6): 443–447. https://doi.org/10.11251/ojjscn.55.443.

17. Hale JE, Darras BT, Swoboda KJ, Estrella E, Chen JYH, Abbott MA, et al. Massachusetts’ findings from statewide newborn screening for Spinal muscular atrophy. *International Journal of Neonatal Screening*. 2021;7(2). https://doi.org/10.3390/ijns7020026.

18. Bitetti I, Manna MR, Stella R, Varone A. Motor and neurocognitive profiles of children with symptomatic spinal muscular atrophy type 1 with two copies of SMN2 before and after treatment: a longitudinal observational study. *Frontiers in Neurology*. 2024;15. https://doi.org/10.3389/fneur.2024.1326528.

19. Nambu Y, Awano H, Bo R, Hong S, Nishio H, Iijima K. Treatment of a 50-day-old Japanese infant with spinal muscular atrophy type 1 using onasemnogene abeparvovec. *No To Hattatsu*. 2022;54(4): 262–265. https://doi.org/10.11251/ojjscn.54.262.

20. Pitarch Castellano I, López Briz E, Ibáñez Albert E, Aguado Codina C, Sevilla T, Poveda Andrés JL. Onasemnogene Abeparvovec Administration via Peripherally Inserted Central Catheter: A Case Report. *Children*. 2024;11(5). https://doi.org/10.3390/children11050590.

21. Strauss KA, Farrar MA, Muntoni F, Saito K, Mendell JR, Servais L, et al. Onasemnogene abeparvovec for presymptomatic infants with two copies of SMN2 at risk for spinal muscular atrophy type 1: the Phase III SPR1NT trial. *Nature Medicine*. 2022;28(7): 1381–1389. https://doi.org/10.1038/s41591-022-01866-4.

22. Day JW, Finkel RS, Chiriboga CA, Connolly AM, Crawford TO, Darras BT, et al. Onasemnogene abeparvovec gene therapy for symptomatic infantile-onset spinal muscular atrophy in patients with two copies of SMN2 (STR1VE): an open-label, single-arm, multicentre, phase 3 trial. *The Lancet Neurology*. 2021;20(4): 284–293. https://doi.org/10.1016/S1474-4422(21)00001-6.

23. D’Silva AM, Holland S, Kariyawasam D, Herbert K, Barclay P, Cairns A, et al. Onasemnogene abeparvovec in spinal muscular atrophy: an Australian experience of safety and efficacy. *Annals of Clinical and Translational Neurology*. 2022;9(3): 339–350. https://doi.org/10.1002/acn3.51519.

24. Pane M, Berti B, Capasso A, Coratti G, Varone A, D’Amico A, et al. Onasemnogene abeparvovec in spinal muscular atrophy: predictors of efficacy and safety in naïve patients with spinal muscular atrophy and following switch from other therapies. *eClinicalMedicine*. 2023;59. https://doi.org/10.1016/j.eclinm.2023.101997.

25. Nanri D, Yuge K, Goto K, Kimura T, Yae Y, Mizuochi T, et al. Onasemnogene Abeparvovec Treatment after Nusinersen in an Infant with Spinal Muscular Atrophy Type 1. *The Kurume medical journal*. 2024;69(34): 255–259. https://doi.org/10.2739/kurumemedj.MS6934008.

26. Favia M, Tarantino D, Cerbo LD, Sabia A, Campopiano R, Pani M. Onasemnogene Abeparvovec: Post-infusion Efficacy and Safety in Patients With Spinal Muscular Atrophy (SMA)—A Fondazione Policlinico Gemelli IRCCS Experience. *Hospital Pharmacy*. 2024;59(1): 39–46. https://doi.org/10.1177/00185787231182562.

27. Toro W, Yang M, Georgieva M, Anderson A, LaMarca N, Patel A, et al. Patient and Caregiver Outcomes After Onasemnogene Abeparvovec Treatment: Findings from the Cure SMA 2021 Membership Survey. *Advances in Therapy*. 2023;40(12): 5315–5337. https://doi.org/10.1007/s12325-023-02685-w.

28. Servais L, Day JW, De Vivo DC, Kirschner J, Mercuri E, Muntoni F, et al. Real-World Outcomes in Patients with Spinal Muscular Atrophy Treated with Onasemnogene Abeparvovec Monotherapy: Findings from the RESTORE Registry. *Journal of Neuromuscular Diseases*. 2024;11(2): 425–442. https://doi.org/10.3233/JND-230122.

29. Bitetti I, Manna MR, Stella R, Varone A. Sequential treatment with nusinersen, Zolgensma® and risdiplam in a paediatric patient with spinal muscular atrophytype 1: a case report. *Acta Myologica*. 2023;42(2–3): 82–85. https://doi.org/10.36185/2532-1900-356.

30. Hammond CK, Oppong E, Ameyaw E, Dogbe JA. Spinal muscular atrophy in Ghanaian children confirmed by molecular genetic testing: a case series. *Pan African Medical Journal*. 2023;46. https://doi.org/10.11604/pamj.2023.46.78.32240.

31. Tosi M, Catteruccia M, Cherchi C, Mizzoni I, D’Amico A. Switching therapies: safety profile of Onasemnogene abeparvovec-xioi in a SMA1 patient previously treated with Risdiplam. *Acta Myologica*. 2022;41(3): 117–120. https://doi.org/10.36185/2532-1900-077.

32. Lee BH, Waldrop MA, Connolly AM, Ciafaloni E. Time is muscle: A recommendation for early treatment for preterm infants with spinal muscular atrophy. *Muscle and Nerve*. 2021;64(2): 153–155. https://doi.org/10.1002/mus.27261.

33. Stettner GM, Hasselmann O, Tscherter A, Galiart E, Jacquier D, Klein A. Treatment of spinal muscular atrophy with Onasemnogene Abeparvovec in Switzerland: a prospective observational case series study. *BMC Neurology*. 2023;23(1). https://doi.org/10.1186/s12883-023-03133-6.
